# Toxicity of Urban PM_10_ and Relation with Tracers of Biomass Burning

**DOI:** 10.3390/ijerph15020320

**Published:** 2018-02-12

**Authors:** Rosette Van Den Heuvel, Jeroen Staelens, Gudrun Koppen, Greet Schoeters

**Affiliations:** 1 Environmental Risk and Health Unit, Flemish Institute for Technological Research (VITO), Boeretang 200, 2400 Mol, Belgium; gudrun.koppen@vito.be (G.K.); greet.schoeters@vito.be (G.S.); 2 Flanders Environment Agency (VMM), Unit Air, Kronenburgstraat 45, 2000 Antwerp, Belgium; j.staelens@vmm.be; 3 Department of Biomedical Sciences, University of Antwerp, 2000 Antwerp, Belgium

**Keywords:** biomass burning, cytotoxicity, interleukin-8, Ames II, oxidative potential, levoglucosan, mannosan, galactosan, PAHs

## Abstract

The chemical composition of particles varies with space and time and depends on emission sources, atmospheric chemistry and weather conditions. Evidence suggesting that particles differ in toxicity depending on their chemical composition is growing. This in vitro study investigated the biological effects of PM_10_ in relation to PM-associated chemicals. PM_10_ was sampled in ambient air at an urban traffic site (Borgerhout) and a rural background location (Houtem) in Flanders (Belgium). To characterize the toxic potential of PM_10_, airway epithelial cells (Beas-2B cells) were exposed to particles in vitro. Different endpoints were studied including cell damage and death (cell viability) and the induction of interleukin-8 (IL-8). The mutagenic capacity was assessed using the Ames II Mutagenicity Test. The endotoxin levels in the collected samples were analyzed and the oxidative potential (OP) of PM_10_ particles was evaluated by electron paramagnetic resonance (EPR) spectroscopy. Chemical characteristics of PM_10_ included tracers for biomass burning (levoglucosan, mannosan and galactosan), elemental and organic carbon (EC/OC) and polycyclic aromatic hydrocarbons (PAHs). Most samples displayed dose-dependent cytotoxicity and IL-8 induction. Spatial and temporal differences in PM_10_ toxicity were seen. PM_10_ collected at the urban site was characterized by increased pro-inflammatory and mutagenic activity as well as higher OP and elevated endotoxin levels compared to the background area. Reduced cell viability (−0.46 < *r_s_* < −0.35, *p* < 0.01) and IL-8 induction (−0.62 < *r_s_* < −0.67, *p* < 0.01) were associated with all markers for biomass burning, levoglucosan, mannosan and galactosan. Furthermore, direct and indirect mutagenicity were associated with tracers for biomass burning, OC, EC and PAHs. Multiple regression analyses showed levoglucosan to explain 16% and 28% of the variance in direct and indirect mutagenicity, respectively. Markers for biomass burning were associated with altered cellular responses and increased mutagenic activity. These findings may indicate a role of biomass burning in the observed adverse health effect of particulate matter.

## 1. Introduction

Ambient particulate matter (PM) can differ both in physical properties (e.g., size, shape, hygroscopicity) as well as in chemical composition, resulting in a complex chemical mixture [1]. Currently, PM regulations are mainly based on mass concentrations and less consideration is given to chemical composition. The chemical composition of particles varies with space and time and depends on the emission sources, the specific atmospheric chemistry and the weather conditions e.g., wind direction. Evidence is growing that suggests particles differ in toxicity depending on their chemical composition [1,2]. Variations in composition may affect the harmful effects on human health [3,4,5,6]. New potential air quality metrics, which are not taken into account with PM mass, have been suggested to evaluate health risks, e.g., elemental and organic carbon (EC/OC) and secondary inorganic aerosols [1,7,8].

A number of studies and reviews have focused on individual components of PM and their linkage with adverse health effect and conclusive evidence of associations have been found e.g., respiratory and cardiovascular effects of metals [9,10].

Recently, there is increasing concern regarding the health impact of biomass burning (e.g., wood smoke) [11]. Wood burning has become popular as a primary or secondary combustion source for households. Measuring campaigns indicated a significant contribution from wood combustion to the total measured particulate matter concentrations [12,13,14,15,16,17]. During biomass combustion significant quantities of known health-damaging pollutants, including several carcinogenic compounds, are released that, in some cases, are comparable to the contribution of traffic [18,19,20,21,22]. The cellulose pyrolysis products levoglucosan, mannosan and galactosan have been used as tracers for combustion of all wood types [23,24]. These cellulose-specific monosaccharide anhydrides (MA) are often used to quantify the contribution of residential biomass combustion and wildfires to ambient PM [25,26,27]. Levoglucosan is not considered toxic and it is excreted unchanged in urine within relatively short time [21,28,29]. Although the interest in the toxicity of biomass smoke and other types of biomass burning is increasing, a rather limited number of toxicological studies has been published. Associations between biomass smoke and respiratory and cardiovascular health effects have been investigated but the outcome is not conclusive [14,30,31,32,33,34,35,36,37,38].

Cell studies provide insights into the toxic effects of PM [39]. Efforts have been made to relate chemical components of PM with toxicological studies. Simultaneous evaluation of PM toxicity and the chemical composition of PM collected in different sites or seasons may help to elucidate the complex relationship between specific constituents of PM and biological effects. Changes in PM chemical composition have been linked to the cytotoxic, pro-inflammatory, genotoxic and oxidative potential of PM in vitro [40,41,42,43,44,45,46].

In 2013–2014, PM_10_ sampling campaigns were initiated in two different areas in Flanders with different pollution pressure: an urban area (Borgerhout) and a background site (Houtem). Collected PM_10_ samples were tested in a panel of biological endpoints. Cellular responses including cell viability and inflammation were evaluated after exposure of bronchial epithelial cells (Beas-2B cell line) to the particle fraction while the organic extracts of the samples were tested for their mutagenic capacity. Beside chemical constituents of PM_10_, the endotoxin level and oxidative potential (OP) of samples were analyzed.

Here, we compared the in vitro biological effects in samples originating from the urban site versus the background site and seasonal effects of the responses. In addition, we investigated the relationships between tracers for biomass burning, measured in the urban area and the biological responses.

## 2. Methods

### 2.1. Site Description

The sampling campaigns were conducted at two locations in Flanders: an urban area (Borgerhout) and a background location (Houtem). The urban site in Borgerhout is considered to be representative for urban traffic background. The site is used as an urban background station for routine PM_10_ monitoring in the framework of the air quality directive (2008/50/EC), but is located near (30 m) to a busy 2 × 2-lane road that connects the inner city with a major highway eastwards (33,500 vehicles day^−1^ in February 2010) [47]. There is a bus stop in front of the entrance of the monitoring terrain. The measurement site in Houtem is generally considered as a rural background site in routine Flemish PM_10_ monitoring framework. The station is located in the middle of the polders 500 m from the French-Belgian border, 2 km from the center of Houtem (700 inhabitants), slightly more than 8 km from the Belgian coast.

### 2.2. PM Sampling

#### 2.2.1. PM Sampling for Toxicity Studies

PM_10_ samples were collected using a high-volume sampler, Digitel DHA-80 (Digitel, Hegnau, Switzerland) with a PM_10_ pre-separator. Particles were collected on Teflon T38 filters (Schleicher and Schuell, Dassel, Germany). The filters were changed automatically after 24 h.

Sampling was conducted in parallel at the urban and background site during the period April 2013–May 2014. The sampling schedule was designed to sample with an interval of 6 days in Borgerhout and 12 days in Houtem, yielding 61 days in Borgerhout and 34 days in Houtem over a 1-year period. In total 95 filters were collected and used for toxicity testing (cytotoxicity, inflammatory and mutagenic capacity, OP). All filters were stored at −20 °C until further handling.

Each 24-h Teflon filter was sectioned into parts. A small part of the Teflon filter was used for OP analyses. Half of the Teflon filter was extracted using Accelerated Solvent Extraction (Dionex ASE, Dionex Benelux, Tienen, Belgium). As a first step of this procedure, each filter was loaded into 11 mL extraction cells. The extractions were performed at a pressure of 140 bar and a temperature of 100 °C. A solvent mixture of 1:1 hexane/acetone (CAS 110-54-3, CAS 67-64-1) was used for extraction. Finally, the extracts were dried under N_2_ and dissolved in 1 mL of 10% dimethyl sulfoxide (DMSO) (CAS 67-68-5). The remaining part of the filters was used for particle collection. Briefly, the collected PM_10_ were extracted from the Teflon substrate into pure methanol by sonication for 30 min. The suspension was evaporated using a rotavapor at 40 °C, 337 mbar and 100 rpm. The suspension was sonicated and transferred to a pre-weighed 1.5 mL conical tube. To enable in vitro testing on equal PM mass concentration, the tube was further evaporated under N_2_ to dryness. The conical tube was subsequently weighted again to determine the weight of the particles. Organic extracts and particle fractions were stored at 4 °C. All samples from the urban and background location were used for toxicity testing. Mutagenicity tests were performed on the organic extracts while particulate samples were used to test cytotoxicity and pro-inflammatory effects in Beas-2B cells. The different assays were performed once on each sample.

#### 2.2.2. PM Sampling for Chemical Characterization

Information on the chemical characterization of PM_10_ at the urban site was provided by the Flanders Environment Agency (Vlaamse Milieumaatschappij (VMM)). A detailed description of PM_10_ sampling in the urban area and the chemical characterization of PM_10_ have been described earlier [48,49]. Briefly, from April 2013 to May 2014, samples were collected every sixth day matching the dates of the sampling campaign described in Section 2.2.1. Samples were collected onto 47 mm quartz filters (Pall Tissuquartz™ filters, 2500 QAT-UP, Sigma-Aldrich BVBA, Overijse, Belgium) using a sequential sampler (Leckel SEQ47/50, Sven Leckel, Berlin, Germany) with PM_10_ inlet, running at 2.3 m^3^/h for 24 h per filter. Filters were weighed before and after sampling in order to determine total PM_10_ mass according to the (stricter) standard for PM_2.5_ (EN 14907:2005). Before and after sampling, filters were conditioned at 20 ± 1 °C and 50 ± 5% relative humidity for 48 h, weighed, left for a further 24 h and re-weighed. The composition of PM_10_ was determined. Punches of 1 × 1 cm^2^ were taken per filter and analyzed for monosaccharide anhydrides (Mas) (levoglucosan, mannosan, galactosan) (Cordell et al., 2014), and elemental (EC) and organic carbon (OC). These components were analyzed for PM_10_ sampled on the quartz filters. The (metal) puncher was manufactured by Sunset Laboratory Inc., Hillsborough, NC, USA.

Levoglucosan, mannosan and galactosan were quantified using a validated gas chromatography mass spectrometry (GC-MS) method described in detail by [50]. Briefly, MAs were extracted from 1 cm^2^ filter punches (spiked with 100 ng of methyl β-d-xylopyranoside as internal standard) by sonication in 1 mL methanol, extracts were filtered, dried then derivatized with MSTFA/1% TMCS for 1 h at 80 °C. 0.5 mL of the derivatization product was analyzed using an Agilent 7890A GC and 5975C MS with CTC-PAL autosampler (Agilent Technologies, Wokingham, UK). Quality control samples were included every tenth sample (100/10 ng/sample of levoglucosan/mannosan and galactosan for summer samples, 500/50 ng/sample of levoglucosan/mannosan and galactosan for winter samples) along with a blank extracted filter sample. Calibration was carried out at the beginning of each batch of analysis and was conducted over the range 5–5000 ng/sample for levoglucosan and 1–500 ng/sample for galactosan and mannosan.

Polycyclic aromatic hydrocarbons (PAHs) in TSP (Total Suspended Matter) (fluoranthene, pyrene, benzo(*a*)anthracene, chrysene, benzo(*b*)fluoranthene, benzo(*k*)fluoranthene, benzo(*a*)pyrene, benzo(*g*,*h*,*i*)-perylene and indeno(1,2,3-*cd*)pyrene) were also provided [51] (VMM 2014). TSP samples were collected every sixth day matching the dates of the sampling campaign described in Section 2.2.1 using a high-volume sampler, Digitel DHA-80 (Digitel, Hegnau, Switzerland) on Glass fiber filters (Ederol type 220/1/60). Briefly, filters were extracted (ASE) with dichloromethane, evaporated using nitrogen, suspended in acetonitrile and analyzed by HPLC (Waters; Zellik, Belgium) (Vydac 201 TP-Colom with acetonitrile-water gradient of 50/50% to 100% acetonitrile in 20 min).

Meteorological conditions such as air temperature, wind speed and precipitation amount were monitored and recorded during the sampling campaigns by VMM and KMI (Royal Meteorological Institute from Brussels, Belgium).

### 2.3. Oxidative Potential (OP)

The method used for the detection of radical oxidant species (ROS) generation capacity of PM was the electron paramagnetic resonance (EPR) spectroscopy. This method is based on the trapping of PM induced hydroxyl radicals (OH^−^) mainly generated via Fenton-type reaction in the presence of H_2_O_2_, using the spin trap 5,5-dimethyl-1-pyrroline-*N*-oxide (DMPO). The analysis was done by RIVM using a direct method without PM extractions from the filters [52,53]. Blank filters were used as background control.

### 2.4. Beas-2B Cell Culture

The Beas-2B cell line was obtained from American Type Culture Collection (ATCC number: CRL-9609, LGC Promochem, Teddington, UK). Cell culture and experimental set-up were performed as described in detail earlier [54]. The cells were cultured in a humidified atmosphere at 37 °C and 5% CO_2_. Beas-2B cells were seeded at 2 × 10^4^ cells per well in 96-well plates (200 µL/well; cell culture area 0.32 cm^2^/well) and allowed to adhere for 24 h. PM samples were resolved in PBS (-Ca/Mg) by overnight shaking to a final concentration of 10 mg/mL. Samples were further diluted in Beas-2B cell culture medium to 1 mg/mL and stirred overnight. All PM samples were stirred before further dilution and addition to the cells. The cells were exposed for 24 h to a concentration range of particles: 100, 50, 25, 12.5 µg PM_10_/mL medium (corresponding to 62.5, 31.3, 15.6, 7.8 μg/cm^2^). Each concentration was tested in six replicate wells. Untreated cells were used as a negative control. Inhibition of cell growth was measured by the neutral red cytotoxicity assay. Cell viability (mean of 6 replica wells) was expressed as percentage viable cells compared to the unexposed control cells (100%).

### 2.5. Interleukin-8

After exposure, the culture medium from the replicate wells was collected, centrifuged (2500 rpm, 10 min) to remove the suspended particles and stored at −80 °C until for further cytokine analysis.

Interleukin-8 (IL-8) release by exposed Beas-2B cells was determined as an indicator of inflammatory potency. IL-8 is a major pro-inflammatory mediator involved in cell attraction. IL-8 was measured in the supernatants using enzyme-linked immunosorbent assay (ELISA) kits (Life Technologies, Carlsbad, CA, USA, IL-8 Human Antibody Pair, Novex^®^). The mean IL-8 concentration (pg/mL) of two replicate wells per PM_10_ concentration was calculated. Results are expressed as the ratio between IL-8 production after PM_10_ exposure and IL-8 levels in non-exposed cells.

### 2.6. Endotoxin

The presence of microbial endotoxin activity in PM_10_ samples was assessed using a quantitative LAL (Limulus Amebocyte Lysate) test kit according to the manufacturer’s instructions (QCL-1000 kit, Lonza, Bornem, Belgium). Endotoxin analyses were performed on suspended PM_10_ samples (100 µg PM_10_/mL) that were used for the in vitro toxicity testing. The mean endotoxin concentration of two replicate wells per PM sample was calculated. Extracts from blank filters were used as negative control.

### 2.7. Ames II

The test was performed with the Xenometrix Ames II Mutagenicity Test kit (Endotell). The Ames II-test, based on bacterial reverse mutations, detects base pair substitutions or frameshift mutations caused by a test item in the Salmonella typhimurium bacterial strain TA98 [55]. The direct and indirect mutagenic potential was determined in the absence and presence of an exogenous activating metabolizing enzyme system (S9 mix). The mutagenic potency of each ASE-extract was tested at a concentration of 20 m^3^ air equivalent (volume inhaled air per day under normal breathing) in triplicate. DMSO was used as a negative control. 4-NQO (4-nitroquinoline-*N*-oxide) 0.5 µg/mL + 2NF (-nitrofluorene) 2.0 µg/mL and 2-AA (2-aminoanthracene) 5.0 µg/mL in the presence of a metabolic activation system were used as positive control. Colonies in each well were counted. An increase over baseline (Mean + SD of solvent control) of ≥2.0 was considered positive and *p*-value ≤ 0.05 (*t*-test) is considered significant. Results were expressed as the number of revertants in function of the mass of PM_10_ or volume of air sampled.

### 2.8. Statistics

For statistical analysis, we used Statistica (version 12.6; Dell Inc., Tulsa, OK, USA) and SAS software (version 9.3) (SAS Institute Inc., Cary, NC, USA). Indicated sampling day (*n*) < 61 (urban area), <34 (rural area) reflect either missing data in PM_10_ characteristic or missing data in biological endpoint.

Concentration-response relationships were assessed by one-way analysis of variance (ANOVA) followed by Dunnett’s post hoc test. Comparison of biological response data between rural and urban sites was done by Factorial ANOVA followed by the Tukey’s multiple comparison test or non-parametric tests (Mann-Whitney U Test). Biological effect data obtained at the highest exposure concentration were used in regression analyses. Correlations between different biological responses and urban PM_10_ characteristics were assessed using nonparametric Spearman rank correlation coefficients (*r_s_*). In addition, multiple linear regression analysis was conducted on the mutagenic response data. Variables with a non-Gaussian distribution were logarithmically transformed. Explanatory variables (PM_10_, PAHs, EC, OC, MAs) were identified by a stepwise regression procedure with the *p*-values for variables to enter in the model set at 0.20, and to stay in the model at 0.05. The statistical significance level was set at *p* = 0.05. Interactions between explanatory variables and temperature and wind speed on the mutagenic response was also considered.

## 3. Results

### 3.1. Cytotoxicity

**Figure 1:**
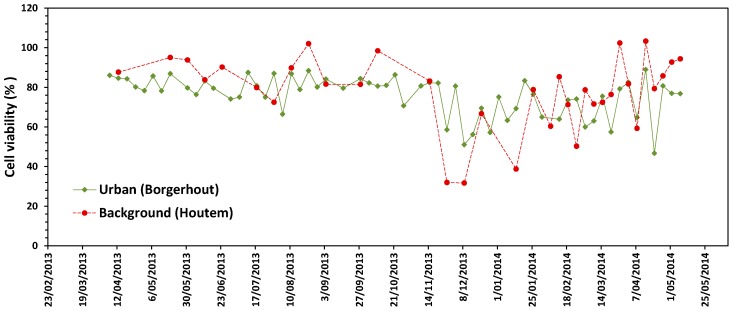
Time series of cytotoxic response of individual PM_10_ air samples between April 2013 and May 2014 for each sampling site. Cell viability after exposure of Beas-2B cells to 100 µg/mL PM_10_ particulate fraction. Cell viability is expressed as percentage viable cells compared to unexposed control cells. Values represent mean of 6 replicate wells.

Human bronchial epithelial cells were exposed to a concentration range of PM_10_ particles collected from the sampled filters from the urban area (urban) and Houtem (rural). The viability of the cells significantly depended on PM_10_ concentration for most samples (87% in the urban area and 82% in rural area) (ANOVA, *p* < 0.01). The average reduction (±95% confidence interval (CI) in cell viability at the highest exposure concentration was 24 ± 2.5% for the urban site and 22 ± 6.2% for the background location. The cytotoxic effect of the PM_10_ fraction was not significantly different between the urban site and background area (Factorial ANOVA, *p* = 0.135). The toxic effect of PM_10_ on cell viability varied from day to day and was highest during the winter months (Figure 1).

### 3.2. Pro-Inflammatory Response

**Figure 2:**
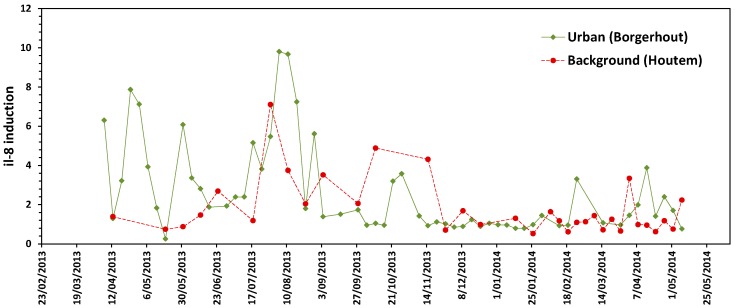
Time series of pro-inflammatory potential of individual PM_10_ air samples between April 2013 and May 2014 for each sampling site. IL-8 induction after exposure of Beas-2B cells to 100 µg/mL PM_10_ particulate fraction. IL-8 induction is expressed as the ratio between IL-8 production after PM_10_ exposure and IL-8 levels in non-exposed cells.

The pro-inflammatory potential of particles as measured by IL-8 induction (ratio PM_10_-exposed vs. negative control) in Beas-2B cells increased significantly with increasing PM_10_ exposure concentration in 30 out of 58 samples from the urban site and in 12 out of 34 samples from the background area (ANOVA, *p* < 0.05). IL-8 induction was significantly higher in the urban area compared to the background location (Factorial ANOVA, *p* = 0.002). The average (±95% CI) IL-8 induction in Beas-2B cells (ratio exposed vs. control) exposed to 100 µg PM_10_/mL was 2.6 ± 0.6 and 1.8 ± 0.5 for the urban and background area, respectively. Daily variations in the pro-inflammatory capacity of particles were seen. The immunological responses were more pronounced in samples collected during spring and summer months (Figure 2).

### 3.3. Mutagenic Response

**Figure 3:**
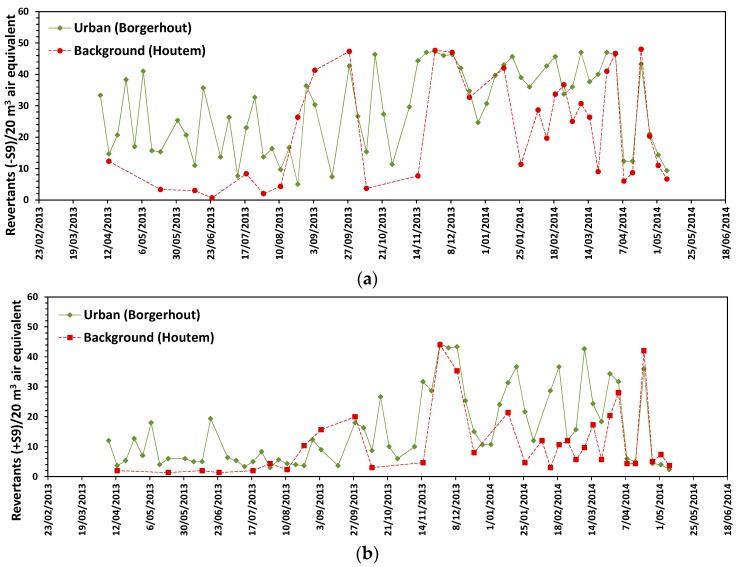
Time series of direct (**a**) and indirect (**b**) mutagenic potential of individual PM_10_ air samples between April 2013 and May 2014 for each sampling site. The mutagenic potency of each ASE-extract was tested at a concentration of 20 m^3^ air equivalent. Mutagenicity is expressed as the number of revertants counting/plate. Values represent mean of 3 replicate wells.

The organic extracts of all urban samples (*n* = 61), assessed at a concentration of 20 m^3^ air-equivalent, showed both direct (−S9) and indirect (+S9) mutagenicity in Ames-II assay whereas 26 out of 31 extracts from the background area had significant positive direct and indirect mutagenic potential. The average number (±95% CI) of revertants in the absence of S9 was 29 ± 3 and 22 ± 6 for the urban and background area, respectively. Mean revertant counts per 20 m^3^ air equivalent (±95% CI) in the presence of S9 were 16 ± 3 and 11 ± 4 for the urban and background area respectively. Both direct (−S9) and indirect (+S9) mutagenic activity were significantly higher in urban samples compared to background samples (Mann-Whitney U test, *p* = 0.04 (−S9) and *p* = 0.03 (+S9)). Day-to-day differences in mutagenic activity were seen in both locations. Direct mutagenicity tended to be higher in samples collected between November 2013 and May 2014 (Figure 3). Direct and indirect mutagenic potential depended on the PM_10_ mass (*r_s_* = 0.46 and *r_s_* = 0.48 respectively, *p* < 0.001).

### 3.4. Endotoxin

Endotoxin concentrations above the detection limit were detected in more than half of the samples and ranged between below the detection limit (<DL) and 8.0 Endotoxin Units (EU)/100 µg PM_10_ in the urban samples and between <DL and 3.1 EU/100 µg PM_10_ in the background samples. The highest concentrations were measured in the summer months of 2013. The median endotoxin concentration was not significantly different between the urban site (0.23 EU/100 µg) and the background area (0.14 EU/100 µg) (Mann-Whitney U Test, *p* = 0.11). A significant positive correlation was seen between the endotoxin concentration and inflammatory capacity of the samples (*r_s_* = 0.77, *p* < 0.001). 

### 3.5. Oxidative Potential

**Figure 4:**
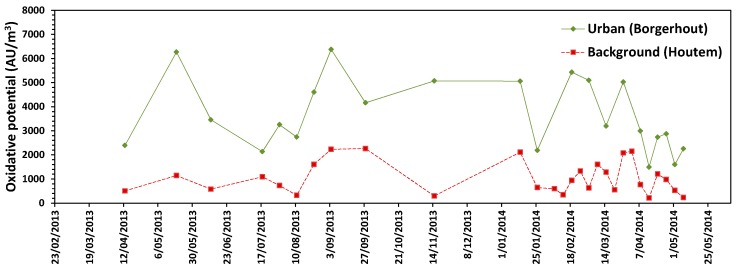
Time series of oxidative potential of individual PM_10_ air samples between April 2013 and May 2014 for each sampling site. OP was measured directly on the filters. Values represent one measurement per filter.

All samples showed oxidative potential (OP) above the detection limit. Day-to-day variations in OP were seen (Figure 4). During the campaign period, the average (±95% CI) OP of urban PM_10_ (3655 ± 660 activity units (AU)/m^3^) was significantly higher compared to the OP in the rural area (1035 ± 257 AU/m^3^) (Mann-Whitney U Test, *p* < 0.01). OP was significantly positively correlated with PM_10_ mass on the filter (*r_s_* = 0.37, *p* < 0.05). OP was not related to the observed cellular responses in Beas-2B cells (cell viability and cytokine induction). On the contrary, OP was associated with direct (*r_s_* = 0.51, *p* < 0.01) and indirect mutagenic activity (*r_s_* = 0.45, *p* < 0.01).

### 3.6. Chemical Characteristics in Urban Air

Detailed information on the chemical composition, temporal variation and source appointment using PMF (Positive Matrix Factorization) of PM_10_ in the urban site Borgerhout has been described by Mooibroek et al. [48]. In a previous study, a selection of the PM_10_ characteristics (BC, As, Cd, Cr, Cu, Mn, Ni, Pb, Zn) from the urban site have been pooled with data from a rural and industrial site to investigate associations between the PM_10_ characteristics and biological effects of PM_10_ [54]. Here, we focus on the role of PAHs and tracers for biomass burning in the observed toxic effects.

**Figure 5:**
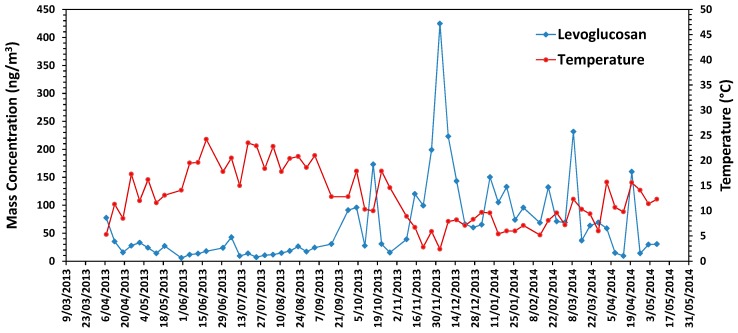
Time series of levoglucosan concentrations and average daily temperature.

**Figure 6:**
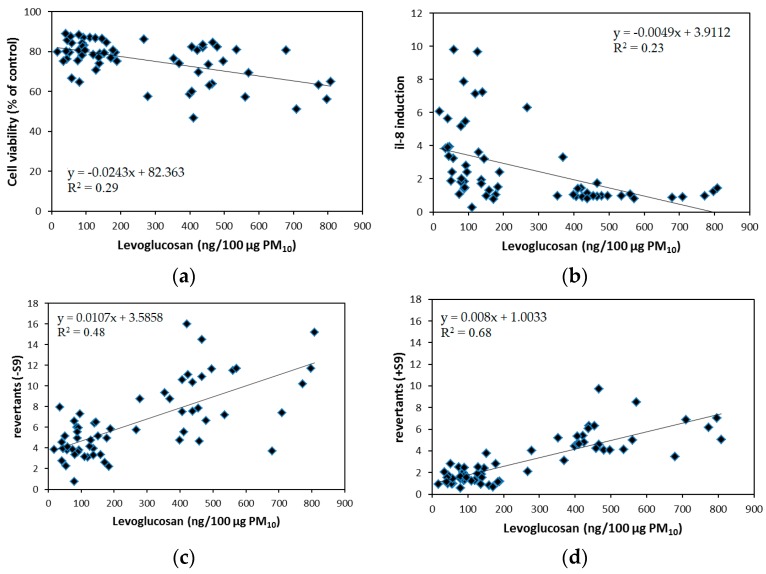
Scatterplots of selected chemical elements with biological responses at an exposure concentration of 100 µg PM_10_. Cell viability (**a**) is expressed as the percentage viable cells compared to the negative control. IL-8 induction (**b**) is expressed as the ratio of IL-8 production in exposed cells compared to unexposed Beas-2B cells. Direct (**c**) and indirect (**d**) mutagenicity are expressed as the number of revertants/100 µg PM_10_-equivalent.

Levoglucosan was the most abundant tracer of biomass burning (Table 1). All three MA tracers were very high inter-correlated (*r_s_* between 0.92 and 0.95, *p* < 0.001) and were most abundant during winter and spring (Figure 5). Moderate correlations (*r_s_* 0.5–0.7) were observed between MA tracers with PAHs concentrations and EC while low correlations (*r_s_* 0.3–0.5) were seen with OC (Table 2). Scatterplots between ambient levoglucosan concentrations and biological responses are shown in Figure 6. Significant positive correlations were seen between direct and indirect mutagenicity and MA tracers (0.55 < *r_s_* < 0.78, *p* < 0.0001) (Table 3). Beas-2B cell viability decreased with increasing concentrations of tracers for biomass burning (−0.46 < *r_s_* < −0.35, *p* < 0.01). Ambient MA tracers showed significant negative associations with IL-8 cytokine induction with *r_s_* ranging between −0.62 and −0.67 (*p* < 0.01). MA tracer concentrations showed no association with OP of the samples.

Mutagenic capacity of the organic extracts correlated moderately with the ambient PAHs levels (Table 3). Indirect mutagenicity correlated with all individual PAHs (*p* < 0.05). Direct and indirect mutagenic capacity of extracts showed the highest correlation with carcinogenic PAHs (*r_s_* = 0.73).

Using multiple regression analyses a significant positive association was found between direct and indirect mutagenic activity of the samples and levoglucosan concentrations which explained respectively 16% and 28% of the total variance in direct and indirect mutagenicity (*p* < 0.001). A significant interaction (*p* < 0.01) temperature and PM_10_ on the direct and indirect mutagenicity was seen.

**Figure 7:**
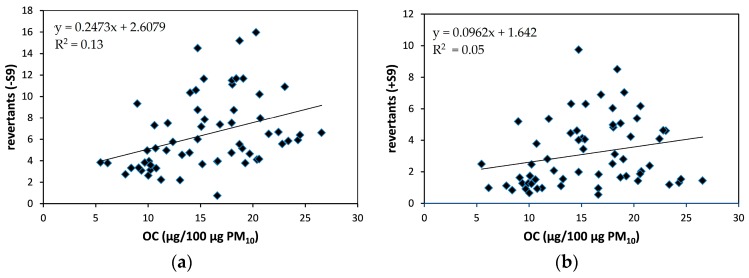
Scatterplots of OC with direct (**a**) and indirect mutagenicity (**b**). Direct and indirect mutagenicity are expressed as the number of revertants/100 µg PM_10_-equivalent.

Both direct and indirect mutagenicity increased with increasing concentrations of EC (respectively *r_s_* = 0.44 and *r_s_* = 0.40, *p* < 0.01) and OC (respectively *r_s_* = 0.49 and *r_s_* = 0.31, *p* < 0.05) (Figure 7). In addition, EC correlated weakly with cytotoxicity (*r_s_* = −0.26, *p* < 0.05) and decreased pro-inflammatory capacity (*r_s_* = −0.26, *p* < 0.05) while a moderate positive association was seen with oxidative potency (*r_s_* = 0.62, *p* < 0.01) of PM_10_.

## 4. Discussion

The present study has focused on the toxicological characterization of PM_10_ collected in an urban site in comparison with samples of a background site. In addition, we provided information on the relationship between the toxicity of urban PM_10_ samples and biomass burning markers in PM_10_.

A concentration-dependent decrease in cell viability induced by the particle fraction of samples collected in both areas was seen but no significant differences in cytotoxicity were found between the two sampling sites.

In order to characterize the particle-induced inflammatory response, IL-8 was measured. Exposure concentrations were chosen to be as low as possible in order to measure inflammatory effects at low cytotoxic doses as cytokine release increases with decreasing cell viability [56,57,58,59]. IL-8 is a major pro-inflammatory mediator involved in cell attraction. Neutrophil recruitment may lead to inflammation. The particle fraction of the samples induced a concentration-dependent increase in inflammatory cytokine induction (IL-8). The increase in IL-8 expression in human airway epithelial cells is related to the activation of membrane TLRs (Toll-like Receptors) and is mediated through an NF-κβ-dependent signaling pathway [60,61]. In vitro effects of particles on cytokine protein release or mRNA production in different cell types has been frequently reported (reviewed by [62,63,64]. In agreement with literature, PM_10_ samples from the urban traffic site induced the strongest inflammatory response compared to the rural site [59,63,65,66,67,68]. These local differences in pro-inflammatory potency cannot be explained by interference from cytotoxicity as particles from different sites caused similar cytotoxic effects. PM_10_ samples collected during warm months were found to have a stronger potency to induce IL-8, confirming other studies [59,65,66,68,69]. The presence of endotoxins in PM might partly explain the pro-inflammatory activity as associations between endotoxin content and the production of cytokines/chemokines in vitro have been reported [44,54,58,65,66,70].

The Ames mutagenicity assay has been used frequently to study the mutagenicity of ambient PM [71]. In the present study, the PM_10_ organic extracts showed mutagenic positive responses. The data showed that direct (TA98 without S9 mix fraction) and indirect-acting mutagens (TA98 in the presence of S9 mix fraction) were present in the samples. The mutagenic responses decreased in the presence of S9 mix, which might indicate that the predominant compounds in the extracts were direct-acting mutagens. This effect has been reported in other studies [72,73,74,75]. Direct-acting mutagenicity can be explained by the presence of nitrated PAH derivates. The results of this study confirm previous observations that mutagenicity tends to increase during the winter seasons [74,76,77,78,79]. Both monitored sites had measurable levels of mutagenic activity. Our data support increased mutagenic activity of PM extracts found in urban areas affected by traffic [41,75,80,81].

Apart from the regional and seasonal differences in biological endpoints, correlations between chemical compounds in urban PM_10_ samples and the biological effects were studied. Although several studies provide some evidence regarding the role of PM components, there is still no unequivocal answer as to which specific components are the most significant determinants of the biological response [82]. Here we focused on the contribution of markers for biomass burning and PAHs to the observed biological responses.

In the current study we found a negative association between the cell viability and the tracers for biomass burning. A reduction in the number of viable cells in human and murine macrophage cell lines by biomass smoke particles has been described in the literature [30,83]. Perrone et al. [43] reported cytotoxicity in A549 cells associated with organic compounds from biomass burning origin.

The inflammatory potential of particles from biomass combustion has not been extensively investigated. The present data showed a significant negative association between IL-8 induction and MAs. In contrast, several studies reported an inflammatory potential of biomass smoke particles in THP-1 cells and A549 cells [43,83,84,85,86] while others found minor inflammatory responses [87]. Negative correlations between monosaccharide anhydrides and the inflammatory activity in mouse RAW 246.7 macrophages were seen [88]. A recent study using a rat alveolar macrophage cell line has not revealed any correlation between levoglucosan levels and inflammatory gene expression [89].

Direct and S9-mediated mutagenicity was correlated with ambient PAHs levels as has been reported before [72]. However, multiple regression analyses showed that beside PM_10_ mass, the biomass combustion tracers in ambient air contributed mainly to the mutagenicity of the samples. The mutagenic potential of biomass smoke particles has been documented earlier [14].

In this study, markers for biomass burning were associated with altered cellular responses and increased mutagenic activity. Probably, the toxic effects cannot be assigned to the MA itself, but rather to other components that are correlated with MAs or by other, in part unknown, fractions unrelated to biomass burning. It has been suggested that biomass smoke exerts its toxic effect by the induction of oxidative stress [42,43,45,46,89,90,91]. Others suggested that the organic fraction of biomass smoke partly accounted for the induced biological effects [84,86,92]. Applying the PMF technique, Mooibroek et al. [48] described profiles based on the chemical characterization of ambient air at four urban background sites including the urban area of this study and an industrial site in North West Europe. A clear biomass burning profiles was found containing almost all available concentrations for the MAs, a small amount of K^+^ and some OC. PAHs levels were not included in the factor analyses. As in our study, reported correlations between levoglucosan and other components showed highest correlation with the PAHs [15]. However, according to Bolling et al. [84] the presence of PAHs would only at least partly explain the effects seen by biomass smoke particles. As MA tracers in this study correlated with PAHs and EC, MA may be a surrogate for other toxic compounds. Moreover, the measured PAHs and EC may come from other sources than biomass burning e.g., vehicular emissions.

Discrepancies in reported results on the effects of biomass smoke in the literature could be due to the use of different model systems and on the other hand may be explained by differences in the chemical composition of particles originating from different combustion conditions [30]. Toxicological studies are a useful tool to evaluate the effects of PM to have a better understanding of the underlying biological mechanisms. However, these studies have some limitations. The in vitro responsiveness to PM may depend on cell line and the contribution of water-soluble vs. insoluble PM-associated chemicals to the toxic effect is yet unclear. In addition, filter sampling, extraction and resuspension can introduce potential unknown artifacts that can affect the chemical composition and further toxic outcome. In addition, the search for explanatory factors for the observed effects involves of a large number of variables, which makes it difficult to draw statistically significant conclusions.

## 5. Conclusions

In conclusion, we have demonstrated that induced biological responses differed between urban and rural PM_10_ samples. Urban samples showed increased pro-inflammatory activity, OP and mutagenic capacity compared to background samples. In addition, tracers for biomass burning were associated with altered cellular responses and increased mutagenic activity. These findings may indicate a role of biomass burning in the observed adverse effects.

Further toxicological research on the impact of biomass burning on human health is essential in order to define the responsible toxic compounds and underlying mechanisms.

## Figures and Tables

**Table 1:** Chemical composition of PM_10_ in the urban site Borgerhout (number of sampling days (*n*), mean ± SD).

		*n*	Mean ± SD
PM_10_	µg/m³	61	25.19 ±12.65
OC	µg/m³	58	3.74 ±1.93
EC	µg/m³	58	1.45 ± 1.03
Galactosan	ng/m³	61	5.79 ± 6.85
Mannosan	ng/m³	61	15.68 ± 18.91
Levoglucosan	ng/m³	61	66.34 ± 73.04
Fluoranthene	ng/m³	35	0.49 ± 0.41
Pyrene	ng/m³	35	0.35 ± 0.41
Benzo(*a*)anthracene	ng/m³	35	0.21 ± 0.38
Chrysene	ng/m³	35	0.23 ± 0.23
Benzo(*b*)fluoranthene	ng/m³	35	0.15 ± 0.20
Benzo(*k*)fluoranthene	ng/m³	35	0.08 ± 0.14
Benzo(*a*)pyrene	ng/m³	35	0.15 ± 0.32
Benzo(*ghi*)perylene	ng/m³	35	0.13 ± 0.19
Indeno(1,2,3-*cd*)pyrene	ng/m³	35	0.09 ± 0.13

OC = organic carbon; EC = elemental carbon; *n* = number of sampling days; SD = standard deviation.

**Table 2:** Spearman rank correlation coefficients (*r_s_*) between MA tracers and OC, and PAHs. Significant correlations are indicated (* *p* < 0.05; ** *p* < 0.01).

	*n*	Levoglucosan	Galactosan	Mannosan
EC	61	0.60 **	0.59 **	0.56 **
Fluorantheen	35	0.47 **	0.44 **	0.39 *
Pyreen	35	0.59 **	0.56 **	0.50 **
Benzo(*a*)anthraceen	35	0.67 **	0.66 **	0.61 **
Chryseen	35	0.58 **	0.59 **	0.54 **
Benzo(*b*)fluorantheen	35	0.67 **	0.63 **	0.64 **
Benzo(*k*)fluorantheen	35	0.68 **	0.68 **	0.63 **
Benzo(*a*)pyreen	35	0.70 **	0.71 **	0.65 **
Benzo(*ghi*)peryleen	35	0.67 **	0.67 **	0.62 **
Indeno(1,2,3-*cd*)pyreen	35	0.57 **	0.57 **	0.52 **

EC = elemental carbon; *n* = number of sampling days.

**Table 3:** Spearman rank correlation coefficients (*r_s_*) between chemical characteristics of PM_10_ and both direct and indirect mutagenicity (variables expressed on mass base (per 100 µg PM_10_-equivalent). Significant correlations are indicated (* *p* < 0.05; ** *p* < 0.01; *** *p* < 0.001).

Chemical Compound	Direct Mutagenicity	Indirect Mutagenicity
**MA** (*n* = 61)		
Galactosan	0.60 ***	0.78 ***
Mannosan	0.55 ***	0.70 ***
Levoglucosan	0.64 ***	0.77 ***
**PAHs** (*n* = 35)		
Fluoranthene	0.18	0.39 *
Pyrene	0.29	0.54 ***
Benzo(*a*)anthracene	0.25	0.60 ***
Chrysene	0.43 **	0.68 ***
Benzo(*b*)fluoranthene	0.25	0.60 ***
Benzo(*k*)fluoranthene	0.42 *	0.69 ***
Benzo(*a*)pyrene	0.45 **	0.72 ***
Benzo(*ghi*)perylene	0.43 **	0.64 ***
Indeno(1,2,3-*cd*)pyrene	0.30	0.53 ***
Sum PAHs	0.38 *	0.63 ***
Sum cPAHs ^$^	0.45 **	0.73 ***
Sum non-cPAHs ^$$^	0.25	0.50 **
**OC** (*n* = 61)	0.47 ***	0.31 *
**EC** (*n* = 55)	0.44 ***	0.40 **

^$^ Sum of carcinogenic PAHs (benzo(*a*)anthracene, chrysene, benzo(*b*)fluoranthene, benzo(*k*)fluoranthene, benzo(*a*)pyrene, indeno(1,2,3-*cd*)pyrene); ^$$^ Sum of non-carcinogenic PAHs (fluoranthene, pyrene, benzo(*ghi*)perylene); MA = monosaccharide anhydrides; OC = organic carbon; EC = elemental carbon; *n* = number of sampling days.
